# The emerging role of glycolysis and immune evasion in gastric cancer

**DOI:** 10.1186/s12935-023-03169-1

**Published:** 2023-12-09

**Authors:** Shanshan Zheng, Huaizhi Li, Yaqi Li, Xu Chen, Junyu Shen, Menglin Chen, Cancan Zhang, Jian Wu, Qingmin Sun

**Affiliations:** 1 Jiangsu Province Hospital of Chinese Medicine, Affiliated Hospital of Nanjing University of Chinese Medicine, Jiangyin Hospital of Traditional Chinese Medicine, Jiangyin Hospital Affiliated to Nanjing University of Chinese Medicine, Jiangsu, China; 2 No.1 Clinical Medical College, Nanjing University of Chinese Medicine, Nanjing, Jiangsu 210023 China

**Keywords:** Gastric cancer, Glycolysis, Immune evasion, Immunotherapy, Microenvironment

## Abstract

Gastric cancer (GC) is the fifth most common malignancy and the third leading cause of cancer-related deaths worldwide. Similar to other types of tumors, GC cells undergo metabolic reprogramming and switch to a “predominantly glycolytic” metabolic pattern to promote its survival and metastasis, also known as “the Warburg effect”, which is characterized by enhanced glucose uptake and lactate production. A large number of studies have shown that targeting cancer cells to enhanced glycolysis is a promising strategy, that can make cancer cells more susceptible to other conventional treatment methods of treatment, including chemotherapy, radiotherapy and immunotherapy, and so on. Therefore, this review summarizes the metabolic characteristics of glycolysis in GC cells and focuses on how abnormal lactate concentration can lead to immunosuppression through its effects on the differentiation, metabolism, and function of infiltrating immune cells, and how targeting this phenomenon may be a potential strategy to improve the therapeutic efficacy of GC.

## Introduction

In the 1920s, Otto Warburg first showed that, unlike normal cells, which catabolize glucose by oxidative phosphorylation (OXPHOS) in the mitochondria, tumor cells tend to convert glucose to lactate even in conditions of sufficient oxygen [1]. This phenomenon was termed aerobic glycolysis or the Warburg effect and is characterized by enhanced glucose uptake and lactate production. Although the adenosine triphosphate (ATP) production efficiency was low during aerobic glycolysis, it still took up to 50–70% of the ATP supply in different tumors [2]. Furthermore, the metabolic intermediates generated during aerobic glycolysis can be used for the biosynthesis of biomacromolecules used by the tumor to meet the demands for rapid growth [3]. The production of lactate also provided an acidic environment to aid the invasion and metastasis of cancer [4].

Gastric cancer (GC) is one of the most malignant tumors worldwide and remains a major health threat in Asia-Pacific regions presently, while its pathological mechanism is generally unknown [5]. Furthermore, despite advances in diagnosis and treatment, prognosis has not improved significantly in the past decade. The five-year survival rate in China is only 35.9%, and the mortality rate remains very high [6]. The occurrence, development, and metastasis of GC are inseparable from its survival environment—tumor microenvironment (TME). A compelling body of research has demonstrated that the stomach has a strongly acidic environment and a unique endocrine system, which is very suitable for promoting tumor progression and metastasis [7]. GC originates within gastric epithelial cells, and similar to other tumor types, it demonstrates a Warburg effect. This involves heightened glucose uptake, intensified glycolysis, and the conversion of substantial pyruvate to lactic acid instead of OXPHOS for energy provision under aerobic conditions [1]. The metabolic alterations primarily driven by the Warburg effect have been termed metabolic reprogramming, enriching our comprehension of tumor cell metabolism. Concurrently, tumor cell glycolysis yields significant lactic acid, resulting in an acidic immunosuppressive microenvironment. This, in turn, exerts metabolic stress on infiltrating immune cells, fostering the development of immunosuppression and immune evasion [8, 9]. Further understanding of strategies to confine these adverse metabolites’ impact on immune cell function could facilitate the adept application of suitable immunotherapies, culminating in optimal therapeutic efficacy.

## Glycolysis in GC

Cancer cells have a significant metabolic difference from normal cells due to the Warburg effect. Song et al. [10] used gas chromatography/mass spectrometry (GC/MS) to analyze the tissue metabolites of GC patients and healthy controls. GC/MS analysis revealed that several intermediates of aerobic glycolysis pathways, such as fumaric acid and alpha-ketoglutaric acid, showed a significant increase in cancer tissue compared to normal mucosa. This suggests that the modified glucose metabolism might serve as a pivotal parameter in delineating GC cells from their normal counterparts. In another investigation, Ikeda and colleagues [11] revealed elevated serum levels of 3-hydroxypropionic acid and pyruvic acid in GC patients. Likewise, abnormal glucose metabolism has been observed in GC tissue by various other researchers [2–4, 12]. Bhattacharya et al. [13] provided evidence that hypoglycemia and heightened glycolysis contribute to increased drug resistance in GC when exposed to chemotherapy. Lin et al [14] further confirmed that TIIA therapy inhibited cell growth and proliferation of GC by inhibiting glucose metabolism of cancer cells.

The association between cancer onset and the activation of proto-oncogenes along with the deactivation of tumor suppressor genes, intricately linked to glucose metabolism, is widely recognized. As a proto-oncogene, Myc plays a significant role in regulating glucose metabolism by increasing the expression of glycolytic enzymes, including glucose transporter 1 (GLUT1) [15], lactate dehydrogenase A (LDHA) [16, 17], and pyruvate Kinase M2 (PKM2) [18]. Simultaneously, the inhibition of p53, a well-known tumor suppressor, directly contributes to the Warburg effect. In various cancer types, the loss of p53 has been observed to promote the flow of glucose through the glycolytic pathway while reducing OXPHOS [18]. The p53 protein enhances OXPHOS while restraining glycolysis by suppressing the expression of GLUT1, GLUT3, and GLUT4 [19], as well as deactivating glycolytic enzymes like phosphoglycerate mutase (PGM) [20]. Compared to normal cells, which generate energy primarily through mitochondrial OXPHOS, cancer cells predominantly obtain energy through increased glycolysis even under aerobic conditions. Conversion of glucose to lactate via glycolysis is inefficient in ATP generation but produces a large number of intermediates that drive cell proliferation. Therefore, increased glucose consumption leading to anaerobic glycolysis is thought to provide an evolutionary advantage for cancer cells [21].

**Fig. 1:**
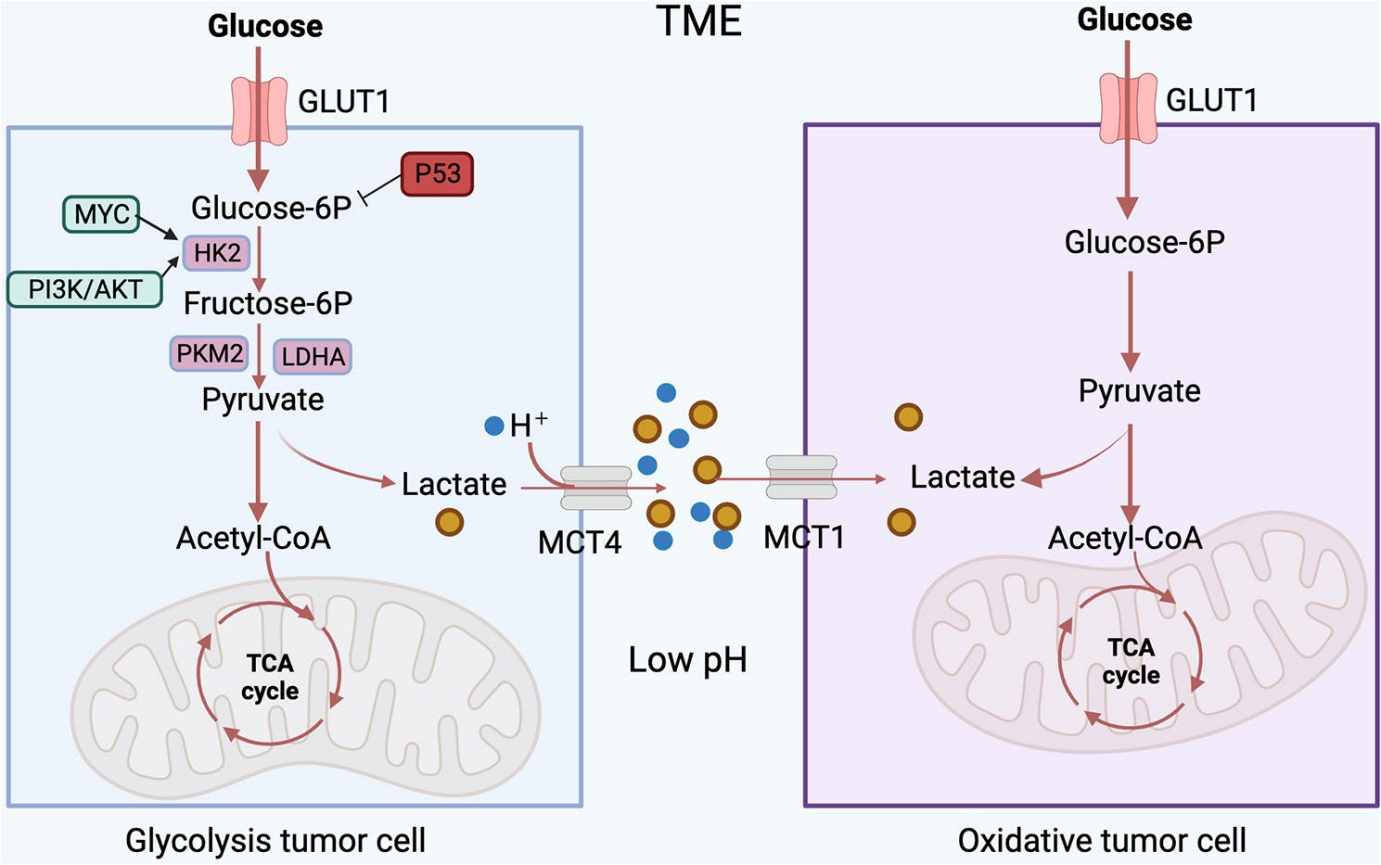
Regulation of the progress of lactate metabolism in cancer cells. Cancer cells, under hypoxic conditions, primarily derive energy through glycolysis while producing excessive lactate, which is exported to the TME through MCT4. The accumulation of lactate allows cancer cells to maintain an acidic microenvironment, shielding them from the effect of therapeutic agents. Additionally, cancer cells near blood vessels are normoxic and can oxidize lactate for ATP synthesis via MCT1. This lactate metabolic symbiosis occurs not only in cancer cells but also in other cells(e.g. immune cells)

 Moreover, several studies have indicated that glycolytic cancer cells offer further advantages for tumor growth by adapting selectively to the tumor TME. For example, extracellular accumulation of lactate from glycolysis alters the TME by creating an acidic pH that is harmful to normal cells. In general, lactate concentrations in normal serum are between 1.5 and 3 mM [22], while in tumor patients they increase to 10-30 mM and can even reach incredibly high levels (50 mM) in the inner tumor cores [23]. Numerous studies have illustrated that elevated lactate concentrations can be absorbed by various cell types and utilized as a metabolic resource within the TME [24–26]. Interestingly, cancer cells, encompassing both glycolytic and oxidative phenotypes, exhibit metabolic heterogeneity contingent upon their spatial distribution within the tumor [27]. Specifically, glycolytic cancer cells are located far from the vasculature, while oxidative cancer cells are located near the vasculature. Lactic acid and protons are co-exported through monocarboxylic transporters (MCTS) [24] to produce extracellular lactic acid. Cancer cells far away from blood vessels are in a state of hypoxia, which mainly obtains energy through glycolysis, and at the same time produces excessive lactic acid, which is exported to TME through MCT4 [28]. Cancer cells situated near blood vessels experience normal oxygen levels and can utilize lactate through MCT1 for ATP synthesis. This symbiotic lactate metabolism is not limited to cancer cells alone; it also involves other cell types like cancer-associated fibroblasts (CAFs) and tumor-associated endothelial cells [29–31]. Additionally, Gladden LB and colleagues demonstrated that inhibiting MCT1 disrupts glycolysis and hampers glutathione synthesis in tumor cells. This disruption elevates intracellular hydrogen peroxide levels, causing mitochondrial damage and eventually cell death [32]. Therefore, the transport of lactate between different cell populations emerges as a significant aspect of the TME, playing a crucial role in tumor initiation, progression, and metastasis (Fig. 1). Excessive lactate fosters the creation of an immunosuppressive environment that nurtures cancer cell growth and significantly influences immune cell functionality [33]. A growing body of research is directed toward unraveling the interplay between lactate and various immune cells within TME, to enhance the effectiveness of ongoing antitumor immunotherapies.

## Glycolysis mediates immunosuppression

**Fig. 2:**
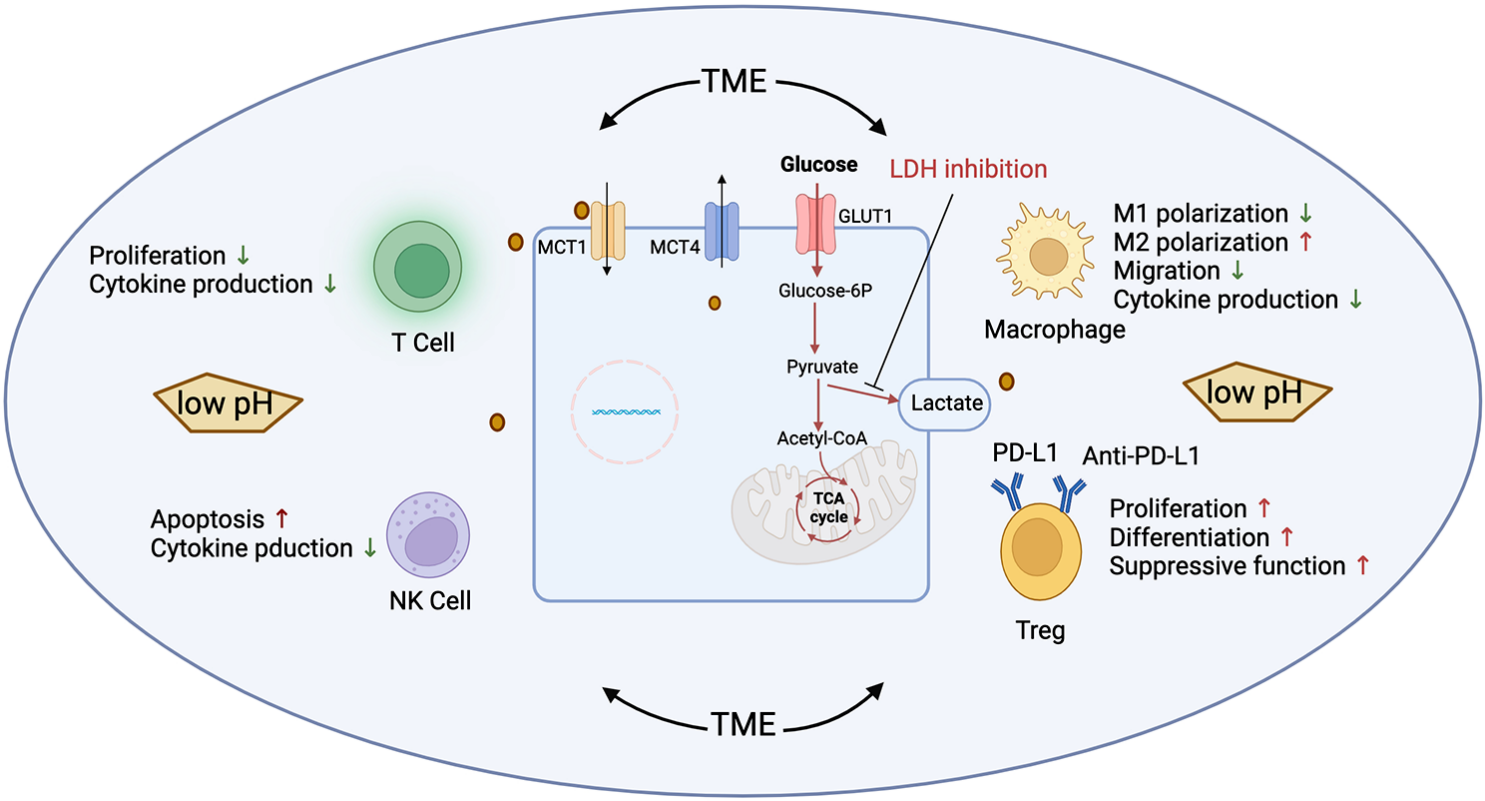
Impact of lactate accumulation on immune cells within the TME. Within the TME, tumor cells predominantly consume nutrients while concurrently releasing excessive lactate, which leads to acidosis and immune suppression. Lactate further influences the metabolic activities of both innate and adaptive immune cells, impeding the functions of T cells, NK cells, and macrophages. Conversely, lactate promotes the sustenance of Treg cells, allowing them to maintain their immunosuppressive functions in the acidic milieu. Combining immunotherapy with medications targeting lactate production and lactate transporters can enhance the therapeutic efficacy of immune checkpoint inhibitors

Although lactate generated by glycolytic cancer cells has been linked to the suppression of anticancer immune cells, the precise mechanism underlying this inhibition has remained elusive. This lack of clarity primarily stems from the fact that immune cells themselves shift to glycolysis and generate lactate during their growth or maturation/activation processes [34, 35]. Consequently, exposure to lactate isn’t entirely novel to immune cells and, in itself, might not exhibit cytotoxic effects on them. Here, we will specifically discuss lactic-mediated antitumor immunity and focus on T cells, NK cells, Treg cells, and tumor-associated macrophages (TAMs). (Fig. 2)

### T cells

T cell-mediated immunity constitutes the central component of the immune response against cancer. Within this category, various T cell subtypes exist, including CD4, CD8, helper T cells, memory T cells, and Tregs. Each subset of T cells possesses distinct functions, encompassing both anti-tumor activities and mechanisms of immune evasion. Among these, CD8^+^ T cells and Tregs are two specific lymphocyte types recognized for their roles in cancer immunity. T cells can perceive extracellular lactate levels, and this sensing process influences intracellular signaling, cellular function, and overall homeostasis. Elevated levels of lactic acid can hinder T cell-mediated immune responses. Within the TME, when the pH falls within the range of 6.0 to 6.5, activated effector CD8^+^ and CD4^+^ T cells typically lose their responsiveness. This results in a reduction in their cytolytic activity and cytokine production [30, 36, 37]. Another study further validated that lactate inhibited the production of interferon (IFN), tumor necrosis factor (TNF), and IL-2 triggered by the T cell receptor (TCR). It also impairs the function of cytotoxic T lymphocytes by suppressing the phosphorylation of the p38 signaling protein [38]. The CD155 molecule present on the surface of GC cells can interact with the immune checkpoint molecule TIGIT, which is found on the surface of T cells. This interaction hampers the glucose metabolism of T cells, diminishes the production of IFN-γ, and suppresses the cytotoxic activity of CD8^+^ T cells [39].

Understanding the regulatory mechanisms of T-cell immunity is crucial for immunotherapy against cancer cells. However, there are still many unknown factors in this complex microenvironment.

### NK cells

As important effectors of host immunity, NK cells induce apoptosis of cancer cells by secreting IFN-γ [40], and TNF-α [41] or forming the Fas/FasL and TRAIL/TRAILR [31] complexes. NKG2D is an important receptor for NK activation, and MICA and MICB are known NKG2D inhibitory ligands that inhibit NK function [42, 43]. Numerous investigations have reported that GC cells can diminish the expression of NKG2D, thereby dampening the activity of NK cells. This is achieved by releasing soluble MICA and MICB molecules. For instance, Midkine has been shown to elevate CHOP expression and create complexes with the transcription factor AP-1. This, in turn, leads to increased MICA/B expression while concurrently inhibiting NK cytotoxicity [44]. STA21 was found to enhance the expression and release of MICB by inhibiting the STAT3 signaling pathway. Consequently, this suppression of STAT3 led to a reduction in NKG2D expression, impairing the function of NK cells [45]. On the other hand, inhibiting matrix metallopeptidase increased the expression of NKG2D ligand, consequently boosting NK activity in GC [46].

High concentrations of lactate have been reported to affect the cytotoxic activity of NK cells by decreasing intracellular pH and inducing apoptosis of NK cells [47–49]. Furthermore, lactate inhibits the activation of nuclear factor-activated T cells (NFAT) in NK cells, thereby inhibiting the production of IFN-γ [50]. Elevated lactate levels not only directly limit the cytolytic functions of NK cells but also indirectly inhibit NK cells by increasing the number of MDSCs [51]. Restoring NK cell function and cytotoxicity is the key to effective treatment of GC, which should be carefully considered in the combined treatment strategy.

### Treg cells

However, not all immune cells in TME react negatively to lactate. Tregs make up about 5 to 10% of the peripheral blood and lymphocytes of humans and mice. Tregs are a potent immune system inhibitor responsible for maintaining immune homeostasis and preventing autoimmunity. The TME actively recruits and promotes the differentiation of Tregs by increasing the expression of forkhead box protein P3 (FOXP3) and MCT1 [52, 53]. The elevated presence of FOXP3 can suppress c-Myc and glycolysis, elevate OXPHOS, enhance NAD(^+^) oxidation, and reconfigure the metabolic profile of Treg cells. This adaptation makes Treg cells more resilient in low glucose and high lactic acid TME [37]. Research has indicated that FOXO4 expression is diminished in GC cells. Reinstating FOXO4 expression notably decreased the glycolysis rate in GC cells, whereas inhibiting FOXO4 expression led to an increase in the glycolysis rate [54]. Furthermore, MCT1-mediated lactating flux and intracellular lactate metabolism are crucial for tumor-infiltrating Treg cells to maintain their suppressive activity, while high glucose levels dampen their function and stability [30]. Furthermore, lactic acid is crucial for tumor-infiltrating Treg proliferation and function [30]. It was found that as the disease progressed, the accumulation of Treg cells in TME gradually increased, resulting in Treg cell imbalance in patients with GC. Furthermore, Treg cells help tumor cells escape host immune surveillance by secreting TGF, which promotes tumor progression [55].

### TAMs

Cancer cells not only evade immune surveillance but also influence the physiology of selective immune cells to promote the rapid growth of tumors. Macrophages are the main component of tumor-infiltrating lymphocytes. TAMs play important roles in angiogenesis, metastasis, and immunosuppression. TAMs can be categorized into two subtypes: M1, also known as classical activated macrophages, and M2, referred to as activated macrophages. M1 macrophages exhibit anti-tumor activity, whereas M2 macrophages promote cancer development. In GC samples, there is a notable abundance of M2-TAMs, and they are strongly linked to invasion, metastasis, peritoneal dissemination, and unfavorable prognostic outcomes [56, 57]. They can change their character according to their environment. Tumor-derived lactate is reported to directly induce cancer-associated macrophages to become M2-like polarized cells, promoting tumor growth in the TME [58]. Mechanistically, the activation of extracellular signal-regulated kinase (ERK)/transcription 3 (STAT3), the activator of the STAT3 signaling pathway and the stimulated expression of vascular endothelial growth factor (VEGF) and arginase-1 (ARG1), as well as the stabilization of the hypoxic inducible factor 1 (HIF-1) contribute to lactate-induced polarization of M2 macrophages and its protumorigenic effects in breast cancer [59, 60]. Furthermore, tumor-derived lactate can induce TAM polarization to the immunosuppressive M2 phenotype by binding to the lactate-sensitive receptor-G protein-coupled receptor 132 (GPR132) [58, 59]. Overall, it is conceivable that the lactate-enriched environment within TME drives the re-education of TAMs to an M2 phenotype. Lactate significantly influences the metabolic reprogramming and immunomodulatory effects of macrophages, chiefly by promoting polarization shifts that detrimentally impact tumor immune responses. In contrast to the glycolytic metabolism that characterizes M1 macrophages, M2 TAMs primarily rely on OXPHOS to fulfill their bioenergetic requirements. Investigators have identified that Yes-associated protein 1 (YAP1) exhibits heightened expression in 5-FU-resistant GC tissues when compared to 5-FU-sensitive GC tissues [61]. Additionally, in GC with elevated YAP1 expression, IL-3 secretion has been observed to induce the polarization of macrophages toward an M2-like phenotype, prompting a GLUT3-dependent glycolytic program. Simultaneously, these polarized M2 macrophages contribute to increased resistance of tumor cells to 5-FU (an antimetabolite known as 5-fluorouracil) by secreting CCL8 and activating phosphorylation of the JAK1/STAT3 signaling pathway [61].

### Endothelial cells and dendritic cells

Endothelial cells (ECs) reside in the innermost layer of blood vessels, regulating local vascular tension and permeability while coordinating with neighboring cells to modulate immune/inflammatory responses and blood supply. Tumor angiogenesis involves the proliferation of a vascular network that supports tumors with oxygen and nutrients. Research indicated that distinct metabolic features of ECs in cancer, and the function of vascular ECs can be modulated by metabolites [62]. ECs primarily generate ATP through glycolysis and rely on glucose for proliferation. This phenotype is driven by signaling pathways adjacent to tumors. For instance, conditioned media from hypoxic glioma cells induced the upregulation of surface GLUT1 in ECs, enhancing glucose uptake [63]. Additionally, signals from tumor-delivered VEGF led to the upregulation of 6-phosphofructo-2-kinase/fructose-2,6-biphosphatase 3 (PFKFB3) in endothelial cells, activating PFK-1, further intensifying the glycolytic phenotype [64]. Lactate was also abundant in the TME, where it triggered tube formation in ECs through HIF-1α-dependent NF-κB activation [65].

Dendritic cells (DCs) are pivotal antigen-presenting cells capable of efficiently capturing, processing, and conveying antigenic information to CD8^+^ T cells. The activation of DCs involves metabolic reprogramming, transitioning from OXPHOS to aerobic glycolysis. Lactate inhibits the differentiation of DCs by inducing IL-10 production and leads to the loss of IL-12 in response to Toll-like receptor (TLR) stimulation [66]. Studies have revealed that lactate enhanced tryptophan breakdown metabolism and kynurenine generation in plasmacytoid DCs and immunosuppressive FoxP3^+^CD4^+^ regulatory T cells, resulting in immune suppression within the TME [67]. Simultaneously, high extracellular lactate concentrations inhibited lactate export by DCs, causing intracellular lactate accumulation, which affects the glycolytic process [68].

Therefore, the accumulation of lactic acid in tumors can inhibit the immune behavior of immune cells through a variety of ways and accelerate the immune escape of tumors.

## Targeting glycolysis combined with immunotherapy in GC

Within tumors, the metabolites present in the TME exert a notable suppressive impact on immune cells, particularly anti-tumor T cells. Consequently, treatments focused on metabolic interventions that regulate glucose metabolism to enhance the TME’s conditions could serve as appealing adjunct therapies in combination with immune checkpoint inhibitors (ICIs). Gaining a deeper understanding of strategies to restrict the influence of these harmful metabolites on immune cell function will aid in optimizing the selection of immunotherapies to achieve maximal efficacy.

Recently, a large number of emerging research has emphasized the importance of neutralization in TME and its influence on the treatment outcome. Systematic and bicarbonate-buffered pH values have been shown to improve TME [69, 70]. More importantly, neutralizing of TME-pH through systemic buffering can enhance the outcome of anti-tumor immunotherapy. [71]. Recently, such neutralization efforts to reverse tumor acidosis have shown improved results in NK cell-mediated cancer therapy [72]. In melanoma patients, high LDH levels indicate a poor response to anti-PD-1 immunotherapy [73]. In a mouse melanoma model, an increase in IFN-γ and granzyme B production in NK cells and CD8^+^ T cells as well as an increase in PD-1 antitumor immune responses to immune checkpoint inhibitors have been reported by blocking LDHA can be observed [74]. Another study indicated that the use of the inhibitor GSK2837808A to suppress LDHA in both patient-derived and B16 melanoma cells can enhance the functionality of T cells both in vitro and in vivo [75]. This improvement contributed to the increased efficacy of immune cells, thereby enhancing adoptive cell therapy. In addition, lactate can also be reduced within the TME through targeted export. MCT 1 has the highest affinity for lactate and can import and export lactate based on a substrate concentration gradient, and the proton cancer MCT4 is expressed at higher levels by highly glycolytic tissues, including tumor cells [76]. In preclinical research, many MCT1 and MCT4 small molecule inhibitors, such as 7ACC2, AR-C155858, syrosingopine, and AZD3965, have been developed. However, only AstraZeneca’s AZD 3965 compound is currently being tested in humans (ClinicalTrials.gov NCT 01791595) [76]. Preclinical trials have shown that the combination of MCT1 inhibitor AZD3965 and anti-PD-1 therapy can reduce lactate secretion into the TME, reduce the infiltration of depleted PD-1^+^ Tim-3^+^ T cells in solid tumors, and improve antitumor immunity [77]. In a mouse model of hepatocellular carcinoma (HCC), overexpression of MCT4 leads to inhibition of CD 8^+^ T cell recruitment and reduced activity [78]. The utilization of an MCT4 inhibitor can efficiently halt the acidification process within the TME and stimulate the expression of chemokine (CXC motif) ligands, specifically CXCL9 and CXCL10, through the ROS/NF-κB signaling pathway. Additionally, in murine models of tumors, the inhibition of MCT4 substantially amplifies the therapeutic efficacy of PD-1 immune checkpoint inhibitors [72]. This suggests that the concurrent administration of MCT4 inhibitors could present a potential avenue for immunotherapy-resistant patients with HCC. Similarly, LDHA and MCT in GC cells express abnormally elevated and poor prognosis in patients with significant correlation [79]. Therefore, we speculate whether inhibition of LDHA and targeting MCT have similar effects on gastric tumors as described above. The research on this aspect, however, the current is less and deep enough.

GC has long been histologically classified into diffuse and intestinal types [80]. Recent studies have suggested potential metabolic differences between diffuse and intestinal subtypes of GC [81, 82]. Compared to the diffuse subtype, the intestinal subtype showed upregulation in glycolysis, which is potentially related to the lower expression of GLUT1 in diffuse gastric cancer [82, 83]. The proteome of diffuse gastric cancer was more enriched in metabolic pathways such as fatty acid metabolism, OXPHOS, and amino acid metabolism [81]. However, these differences are not fully elucidated at present, and further in-depth research is required to validate these potential metabolic disparities and determine their significance in the treatment of GC.

To date, glycolysis inhibitors are still in the preclinical stage, so their effect in humans is unclear. Currently, only a few therapeutic studies target GC metabolism. Further research is expected to investigate the role of cancer-specific glycolytic inhibitors in the development of effective therapeutic regimens for GC.

## Conclusion

Modified glucose metabolism stands as a hallmark of GC, offering novel perspectives into GC development and the recognition of biomarkers targeting distinct metabolic facets of the condition. This review primarily focuses on how aberrant lactate concentrations influence tumor infiltration via diverse pathways of immune cell differentiation, metabolism, and function. The discovery of targets related to lactic acid metabolism in cancer immunomodulation has opened up new avenues for immunotherapy. A comprehensive understanding of how lactic acid metabolites influence the functionality of immune cells holds the potential to significantly enhance the effectiveness of immunotherapy. Despite some current obstacles in the clinical application of changes in lactic acid metabolism in immunotherapy for GC, it is anticipated that further in-depth research in these areas will facilitate the clinical utilization of its metabolites in GC treatment in the near future.

## Data Availability

The datasets used and/or analyzed during the current study are available from the corresponding author upon reasonable request.
